# London Surgeons

**Published:** 1864-03

**Authors:** 


London Surgeons.—In a letter to the *American Medical Times*, Prof. Charles A. Lee says: "The London surgeons operate more fearlessly and with more rapidity than ours do on our side of the Atlantic; but I very much doubt whether more successfully, except in particular cases. Thus I saw Mr. Ferguson operate for double cleft palate last week, and the operation was completed in fifteen minutes. He afterwards informed me that the average duration of the operation of staphyloraphy in his hands was ten minutes, and that out of 105 cases, he had met with complete success in 102. This must be admitted to be extraordinary activity and marvellous success. But much of this success is owing to previous frequent manipulations by the finger of the patient, or a tooth brush, of the fauces and parts adjacent, and to the very free separation of the velum palati from the bone, so as to allow great distension. The profession is indebted to Mr. Ferguson for this practice, which he introduced many years ago, but has recently been claimed by another surgeon as having originated with him.

